# Recent Advances in Targeting Tumor Energy Metabolism with Tumor Acidosis as a Biomarker of Drug Efficacy

**DOI:** 10.4172/1948-5956.1000382

**Published:** 2016

**Authors:** Paul J Akhenblit, Mark D Pagel

**Affiliations:** 1 Cancer Biology Graduate Interdisciplinary Program, University of Arizona, Tucson, AZ, USA; 2 Department of Medical Imaging, University of Arizona, Tucson, AZ, USA

**Keywords:** Tumor metabolism, Tumor metabolism inhibition, Tumor acidosis

## Abstract

Cancer cells employ a deregulated cellular metabolism to leverage survival and growth advantages. The unique tumor energy metabolism presents itself as a promising target for chemotherapy. A pool of tumor energy metabolism targeting agents has been developed after several decades of efforts. This review will cover glucose and fatty acid metabolism, PI3K/AKT/mTOR, HIF-1 and glutamine pathways in tumor energy metabolism, and how they are being exploited for treatments and therapies by promising pre-clinical or clinical drugs being developed or investigated. Additionally, acidification of the tumor extracellular microenvironment is hypothesized to be the result of active tumor metabolism. This implies that tumor extracellular pH (pHe) can be a biomarker for assessing the efficacy of therapies that target tumor metabolism. Several translational molecular imaging methods (PET, MRI) for interrogating tumor acidification and its suppression are discussed as well.

## Introduction

Traditional chemotherapeutic agents target cancer cells by inhibiting major pathways involved in rapid cellular division [1]. These include agents that directly damage the DNA or inhibit DNA repair, inhibit DNA or RNA synthesis, or inhibit microtubule formation or function. More recently, targeted therapies have been introduced to specifically target unique phenotypes in cancer, such as particular growth signals and pathways not present in normal cells [2]. For instance, tyrosine kinase inhibitors (e.g. imatinib) are efficacious towards chronic myelogenous leukemia by inhibiting cellular growth signals [3].

While agents that cause DNA damage and arrest mitotic or cellular growth pathways are well explored, traditional chemotherapy agents infrequently inhibit tumor metabolic pathways [4]. First described by Otto Warburg in 1924 and therefore known as the Warburg effect, cancer cells employ altered cellular energetics as their means towards energy production while maintaining biomass production [5]. Even in the presence of oxygen, cancer cells have highly upregulated anaerobic glycolytic pathways which produce lactic acid as a result, and shuttle metabolic precursors into other pathways [6]. This unique metabolism profile presents itself as a target for anti-cancer therapies. Metabolism-targeting therapies are being intensely researched as means for managing tumors and increasing patient survival.

Altered cancer metabolism was originally thought to be the result of an increased need for biomass synthesis that provides a strong growth advantage. However, other purposes of altered cellular energetics have been elucidated [7,8]. The reductive/oxidative balance of the intracellular space is inherent to cellular metabolism. Oxidative phosphorylation generates reactive oxygen species (ROS) which are toxic to the cell when left unbalanced by anti-oxidants [9]. The aberrant glycolytic metabolism, recycling of NADH, and lactate dehydrogenase enzyme activity allows cancer cells to avoid this ROS damage. These mechanisms allow the cancer cell to meet high energy demands without resulting in oxidative stress.

Recent work from the last two decades has shown that altered metabolism pathways can drive carcinogenesis as well instead of sustaining it. As an example, a mutated form of isocitrate dehydrogenase in the citric acid cycle has been shown to drive tumor formation and progression in glioblastomas [10]. Other research has elucidated a link between attenuated mitochondrial function and dependency on glycolytic activity to varying degrees in different cancers, suggesting some cancers rely exclusively on Warburg effect metabolism while others are more flexible [11].

Further research suggests a “chicken and egg” question exists between genomic instability and deregulated cellular energetics. Broadly defined as oncogene theory, 45 the original paradigm argued that cancer biology is driven by loss or gain of function mutations in proto-oncogenes [12]. The school of thought has since grown to describe carcinogenesis beyond proto-oncogenes, and can be wholly summed as the genomic instability [13]. Recent work has demonstrated that transplanting mitochondria from cancer cells into normal cells causes a transformation process without the presence of oncogenes [14]. Therefore genomic instability, oncogene theory, and deregulated energetics together orchestrate carcinogenesis. The potential to inhibit tumor metabolism would provide great benefit to survival in the clinic [15]. Aggressive phenotypes with high rates of mutations, cancer stem cell-ness, or dormant metastatic sites are all suggested or hypothesized to contribute to the difficulty of total treatment and remission [16]. Curative therapies relying on single agents are rare and remain elusive. Similarly, approaches that target cancer metabolism will unlikely provide a single agent with curative mechanics across multiple tumor models. Adaptive, compensatory, and drug resistance mechanisms are possible outcomes in treatments that target metabolism. However, these mechanisms are independent of the genotoxic effects of chemo-, radioand receptor-targeted therapies. Thus metabolism-targeting agents provide an outstanding opportunity for combination with traditional chemotherapies, as metabolism can be targeted simultaneously with mitotic and cellular growth pathways.

The expanding field of molecular imaging is complimentary to anti-cancer drug research and development. Present morphological imaging modalities are used in the clinic to temporally monitor changes in tumor size to determine therapeutic effect. Novel molecular imaging techniques are being developed to help a clinician to better detect and characterize tumors via molecular biomarkers. One such biomarker is tumor acidity, a manifestation of the lactic acid overproduction due to the altered metabolism in cancer [17]. Because the tumor extracellular pH (pHe) is linked to the tumor metabolic activity, it is a great candidate for measuring anti metabolism targeting agents. There are several non-invasive technologies being developed and tested to measure tumor pH, using magnetic resonance imaging or positron emission topography. This review surveys the broad field of metabolism-targeting agents that are either in clinical trials or in pre-clinical development with strong clinical promise, whose efficacy could be potentially assessed by imaging methods that measure tumor acidosis.

## Targeting Energy Metabolism in Cancer

Tumor metabolism has proven to be an extremely plastic process. Cancers are often able to respond with an adaptive feedback to overcome the inhibition of most metabolic targets or general growth signals. Similar to the combination cocktails in chemotherapy (i.e. R-CHOP, RICE), anticancer metabolic treatments will likely be comprised of several agents meant to disrupt metabolic pathways in parallel or antagonistically, such as simultaneously inhibiting the anaerobic respiration pathway and the oxidative phosphorylation pathways.

There are many possible targets for manipulating or inhibiting cellular metabolism, however there are several targets and pathways that have received the most attention and hold clinical promise. We will examine the pathways that have promising agents in modulating tumor metabolism.

### Glucose and fatty acid metabolism pathways

**Figure 1:**
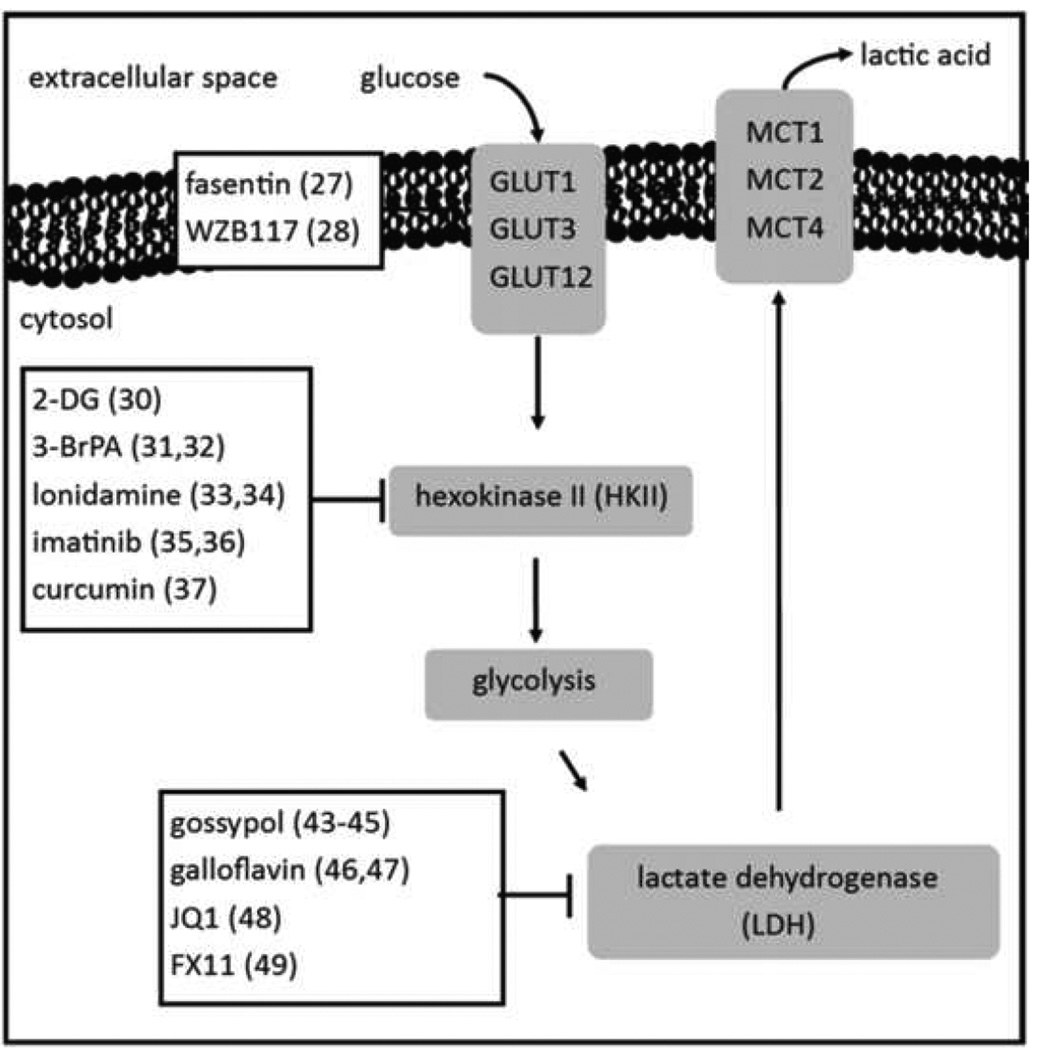
Glycolysis provides cancer cells with energy and biomass production. Most therapies target either hexokinase and its isoforms (hexokinase II being mostly overexpressed in cancer) and lactate dehydrogenase. Targeting the rest of the glycolytic enzymes could be dangerous to the patient as those enzymes are universally expressed. Being the fundamental source of carbon for lactic acid production, inhibiting glycolysis could be assessed by measuring changes in tumor extracellular pH.

Perhaps the most obvious and immediate targets for disrupting cancer metabolism are the enzymes involved in the metabolic breakdown of glucose (Figure 1). There have been some promising steps taken in inhibiting the cell’s ability to metabolize glucose, and inhibiting one or more of these enzymes would also likely suppress tumor acidosis. GLUT1 is the primary transporter of glucose that is under pre-clinical investigation as an anti cancer target [18]. Of the other glucose transport family members, GLUT1 is the most prevalent in cancers, while GLUT3 and −12 have also been found to be expressed in some cancers such as prostate and breast [19,20]. Currently the most promising treatment in pre-clinical inhibition is fasentin [21]. While fasentin has a Fas-based mechanism of action to induce apoptosis, it has been shown to inhibit glucose uptake. Currently no research on fasentin *in vivo* has been reported. A second small molecule, WZB117, showed efficacy in an A549 lung model, which was able to reduce intracellular ATP and glycolysis levels in cancer cells and inhibited tumor growth in a mouse model [22]. The first enzyme in the glycolysis pathway is hexokinase, an enzyme that exists as types I–IV. Hexokinase II (HKII) is the isozyme typically found in neoplasias, and can be either free-floating in the cytosol or bound to mitochondria [23]. The most common inhibitors are 2-deoxyglucose (2-DG), 3-bromopyruvic acid (3-BrPA), and lonidamine, the latter of which shows evidence of inhibiting mitochondrially-bound HKII [24–26]. Somewhat surprisingly, imatinib has shown anti tumor activity as a metabolic inhibitor even though this drug is the default treatment for chronic myelogenous leukemia [27]. Imatinib has been shown to inhibit HK and also glucose-6-phosphate dehyodrogenase (G6PD), a pentose phosphate pathway enzyme whose function is to regulate NADPH levels in T47D and Hep G2 cells *in vivo*. Curcumin was also recently shown to inhibit aerobic glycolysis in colorectal cancers by both downregulating and inhibiting HKII expression and activity in colorectal models *in vitro* [28]. Imatinib, 2DG, and curcumin are all shown to have been safe to use in human studies. Recent work demonstrated 3-BrPA can be administered via microencapsulation with no lethal toxicity in an orthotopic mouse model of pancreatic ductal adenocarcinoma [29].

In normal oxidative phosphorylation respiration, pyruvate is converted to acetyl-CoA by pyruvate dehydrogenase (PDH). However in the Warburg effect, pyruvate is converted to lactic acid by the enzyme lactate dehydrogenase (LDH). Clinically, the LDH levels in the blood are used as a negative prognostic marker, where the LDH found in blood is from the lysis of tumor cells [30]. Because LDH is a chief component of lactic acid production, and acidic tumors are found to be more aggressive and invasive, it would make sense that the negative prognosis comes from tumors with acidified microenvironments. The primary isoform of LDH found overexpressed in most aggressive cancers is LDHA [31]. Because LDHA is the final step in the process of producing lactic acid it is a favorable target as there are no pathways that could circumvent its inhibition and continue producing lactic acid. Additionally, recent studies have identified the “Reverse Warburg effect”, a mechanism by which cancer cells induce aerobic glycolysis in stromal cells, and then the cancer cells internalize the resulting pyruvate in the surrounding parenchyma [32]. This pyruvate is then oxidized in the mitochondria, which generates energy via oxidative phosphorylation, in cancer cells that employ this oxidative pathway. Importantly, the Reverse Warburg effect can only provide energy in cancer cells that have not lost the oxidative phosphorylation pathway. While other glycolytic targets could hamper the cancer’s direct production of pyruvate, the Reverse Warburg effect circumvents the effect by supplying pyruvate to LDH. Therefore, inhibiting LDH can also target the Reverse Warburg effect.

Some LDH inhibitors have been identified and tested clinically. One such example is gossypol, originally investigated as a male sterility agent [33]. However it exhibits calcium blocking properties and can result in paralysis, therefore its use in the clinic is unlikely. A newer LDH inhibitor called galloflavin is being investigated as an LDHA inhibitor, yet no *in vivo* studies to date have been performed [34]. JQ1 has been identified as an LDHA inhibitor and was shown to suppress tumor growth in orthotopic mouse models of ovarian cancers [35]. A fourth candidate is FX11, shown to inhibit LDHA activity and slow tumor growth *in vivo* with a flank tumor model of P493 lymphoma [36]. The rest of the glycolytic enzymes are not typically examined as therapeutic targets. However, agents capable of disrupting the conversion of glucose into lactic acid would likely result suppressing tumor acidosis.

In normal physiology cells direct excess glucose towards fatty acid production by means of fatty acid synthase (FAS), a multi-enzyme construct that creates palmitic acid for use in forming lipid bilayers. Cancer cells have upregulated this metabolic pathway to sustain high levels of cellular proliferation [37]. Evidence shows that targeting FAS in cancer cells slows proliferation as a result from the deficit of available lipids for membrane formation. Cerulenin is a potent FAS inhibitor in multiple myeloma, and the C75 inhibitor was shown to inhibit FAS in HL60 cells [38]. A recently developed inhibitor called TVB-3166 was shown to have anti-cancer properties in various *in vitro* and *in vivo* xenograft models, including COLO-205 and HT-29 cells [39]. Cellular proliferation and metabolism are inherently linked, and therefore targeting FAS would signal for lower energy demand from the cell, resulting in a decrease in tumor extracellular acidification. More importantly, cancer cells can also employ β oxidation of fatty acids, known as fatty acid oxidation (FAO), to produce acetyl-CoA for the TCA cycle. FAO was shown to be the predominant form of energy production in prostate cancers, as well as provide survival to cells that have lost attachment [40]. Recently, carnitine palmitoyltransferase 1 (CPT1) expression in cancer cells was shown to promote FAO and ATP production, tumor growth, rescue from metabolic stress, and resistance to mTOR complex 1 inhibitors [41]. This makes CPT1 a promising target for therapeutic intervention. The last enzymatic step in FAO is catalyzed by 3-ketoacylthiolase (3-KAT), which is also under investigation as a potential new therapy. Trimetazidine is approved for human use in Europe and Asia as a 3-KAT inhibitor. Ranolazine is also approved human use as 3-KAT inhibitor in Europe and the USA, and perhexiline is approved for human use as a CPT1 inhibitor in Australiaand Asia [36]. Removing FAO as a substrate for TCA, and therefore LDHA activity, could lower tumor extracellular pH.

### PI3K/AKT/mTOR signaling pathway

**Figure 2:**
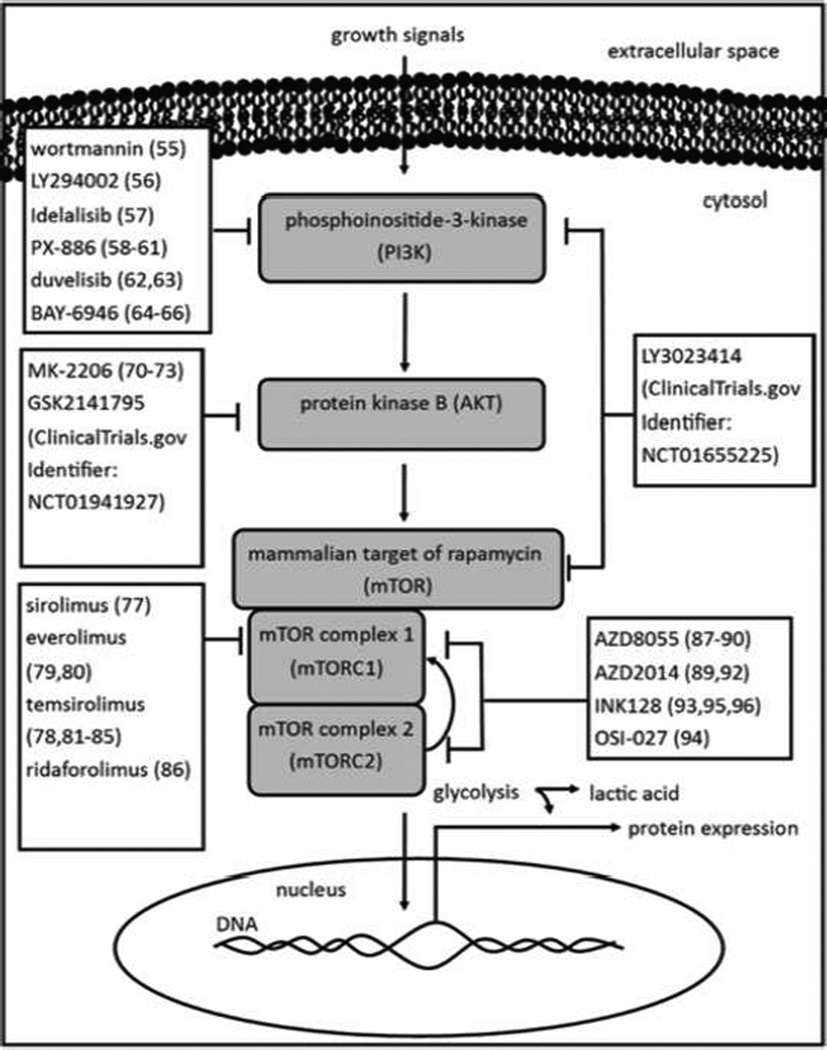
The phosphoinositide-3-kinase/protein kinase B/mammalian target of rapamycin (PI3K/AKT/mTOR or PAM) pathway promotes cell growth/proliferation and cell survival, and sustained metabolic activity. Many therapies exist to target the PAM pathway, however feedback loops intrinsic to the pathway make single target/single agent treatments less efficacious. Dual targeting therapies are gaining momentum in investigations. Because PAM activity regulates glycolysis, measuring PAM targeting drug efficacies by interrogating the tumor extracellular pH is possible.

The phosphoinositide-3-kinase/protein kinase B/mammalian target of rapamycin (PI3K/AKT/mTOR) pathway is a well understood system that is focal towards driving or arresting cellular proliferation in response to growth factor signals (Figure 2). Its responsibility in regulating proliferation also means the pathway modulates biosynthesis signals in the cell. Activating this pathway increases cell growth and division, which in turn activates an increase in metabolism that addresses the need for energy production and biomass production. Inhibiting this pathway can then result in lower energy production, as evidenced by a mTOR inhibition study in which lactic acid production decreased in GBM cell lines, which was correlated with decreased cell proliferation [42]. Therefore PI3K/AKT/mTOR (PAM) inhibitors are also suspected of indirectly inhibiting general tumor metabolism and therefore suppressing tumor acidosis. Similar to the glycolytic pathway, the PI3K/AKT/mTOR pathway 175 presents with a target at each step, and therefore inhibitors for each step are being actively explored. However, the PI3K/AKT/mTOR pathway is not universally dysregulated across all cancers, and PAM inhibitors are only effective in cancers that rely on the PI3K/AKT/mTOR pathway as a driver [43,44]. This allows for tumor acidosis to be used as a biomarker for the efficacy of a PAM inhibitor; if no change in tumor pHe is detected, it is likely the PAM inhibitor will have limited effect and another therapy should be tried. If a larger change in pHe is detected, this result may likely mean that treatment was efficacious installing the tumor metabolism and cellular proliferation. A weakness of PAM inhibitors is that these drugs are often isoform-specific for the common mutations and expression patterns of their targets, thereby limiting their efficacy across different cancers (i.e. glioblastomas versus renal cell carcinomas). As an advantage, isoform specificity also reduces overall toxicity to the patient.

The drugs wortmannin and LY294002 are the most common PI3K inhibitors, however their high toxicity greatly limits their use in the clinic; wortmannin can be fatal and LY294002 can cause off target side effects [45,46]. Currently there exists a long list of PI3K inhibitors in early stage clinical trials, but only a handful of late stage phase II and III trial drugs are being examined. Of the late stage investigations, most promising is idelalisib which demonstrated significant progression free survival in 220 randomized patients with chronic lymphocytic leukemia (CLL) (ClinicalTrials.Gov Identifier: NCT01539512) [47]. Another promising pan-PI3K inhibiting agent under phase II investigation is PX-866, which has been shown to have anti-tumor activity *in vivo* against multiple GBM lines, but did not meet statistical significant endpoints in human trials [48–50]. Other agents in phase II trials include duvelisib in patients with CLL (stable disease state) and BAY 80-6946 in various *in vitro* breast models (BT-20, BT-747, ZR-75-1, MDA-MB-468) resulting in decrease tumor size, however both are isoform specific [51,52]. Lastly, dual PI3K-mTOR inhibitors also being developed as PAM inhibitors, though few molecules are investigated in clinical trials. LY3023414 is one such dual PI3K-mTOR inhibitor being investigated in prostate and non-small cell lung cancers in a phase I clinical trial (ClinicalTrials.gov Identifier: NCT01655225). AKT inhibition is still being studied in phase I and II clinical trials. MK-2206 is currently being studied in phase II across several advanced lung and ovarian solid tumors [53,54].

The drug GSK2141795 is also currently being investigated in phase II studies in melanoma patients, in combination with other therapies (ClinicalTrials.gov Identifier: NCT01941927). With the discovery of rapamycin, the race began to create a mTOR inhibitor for cancer treatment. It was quickly learned that traditional rapalogues were efficient in blocking mTOR complex 1 (mTORC1) activity, but failed in suppressing mTOR complex 2 (mTORC2) activity [55]. A negative feedback loop between mTORC2 and AKT was identified, and second generation mTOR inhibitors now target mTORC1/2. Currently, sirolimus, everolimus, temsirolimus, and ridaforolimus are all investigated as agents in various cancers as mTORC1 inhibitors [56–59]. The molecules AZD8055, AZD2014, INK128, and OSI-027 are under clinical investigation as mTORC1/2 inhibitors in breast, B-cell lymphoblastic leukemia, and colon cancer models [60–63]. Their efficacy as mTOR inhibitors, and therefore metabolism inhibitors, may be assessed with changes in tumor pHe.

### Hypoxia-inducible factor pathway

**Figure 3:**
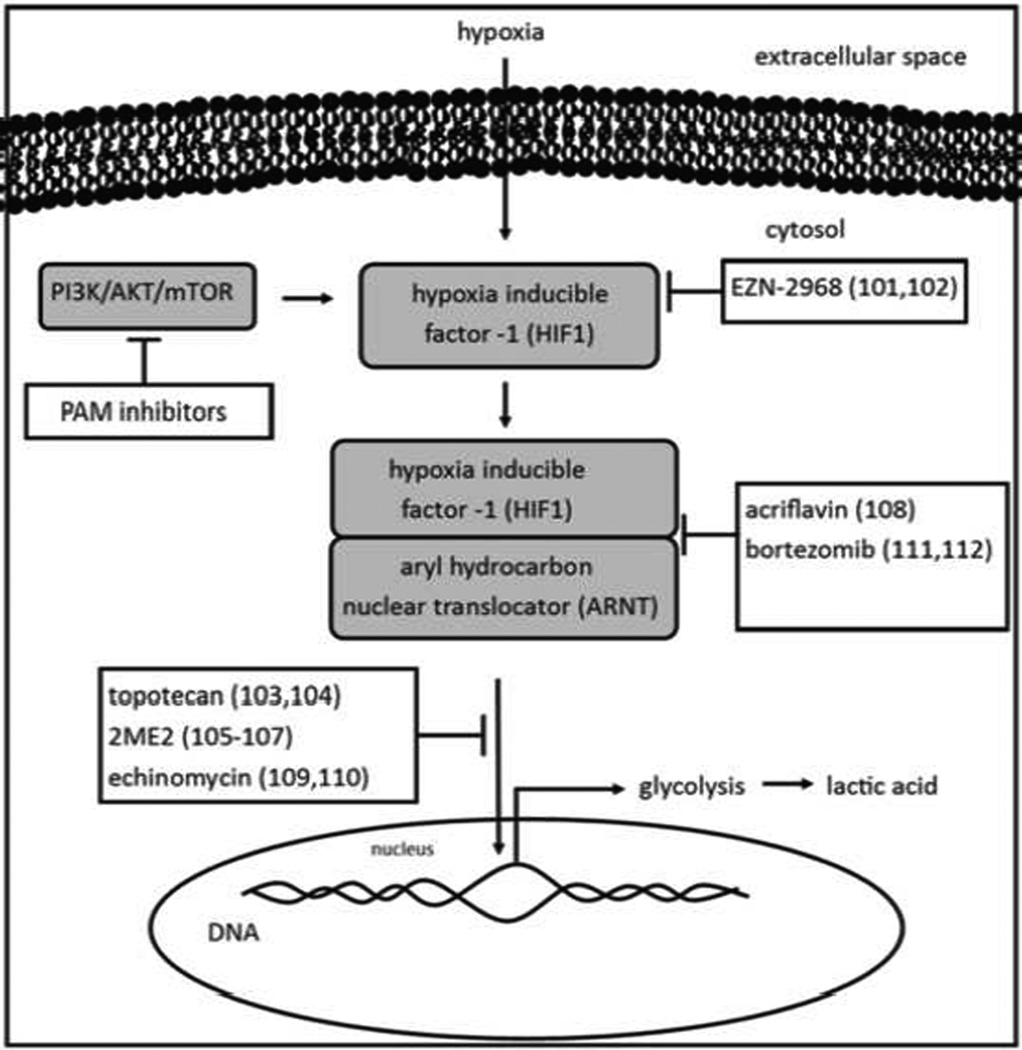
Tumors inevitably outgrow their blood supply, resulting in HIF-1 activation as a result of the hypoxic microenvironment. HIF-1 regulates glycolysis, and because it is not expressed at elevated levels in normal tissues, makes for a good candidate for targeting tumor metabolism. HIF-1 regulates glycolysis, the principle source of carbon for tumor acidosis. Therefore HIF-1 targeting therapy efficacy can be measured by assessing changes in tumor extracellular pH.

The microenvironment of the tumor and its internal biology are inherently intertwined. The tumor reacts to its microenvironment by selecting for phenotypes that have advantages in that space, and can also change the microenvironment to its own needs with those same phenotypes. An example of this relationship is the cycle of breast cancer metastases in the bone and their osteolytic/osteoblastic signaling pathways. The metastasized cancer cells provide stimulus for osteoclast or osteoblast activity, which in turn provides the cancer cells with additional growth factors. Likewise, cancer cells activate the hypoxia-inducible factor 1 family (HIF-1) transcription factors in response to poorly oxygenated environments (Figure 3). HIF-1 turns on angiogenesis and activates anaerobic glycolysis in hypoxic microenvironments [63]. HIF-1 activity leads to acidic tumor microenvironments, so inhibiting HIF-1 from activating its downstream transcription targets could arrest the progression towards the acidification of the tumor space. Additionally, HIF-1α activation up regulates vascular epithelial growth factor (VEGF) expression, which manifests as the angiogenic hallmark in tumor biology. With less vasculature, less oxygen and nutrients are delivered throughout the tumor, decreasing the metabolic rate of the tumors by limiting substrates. Therefore interfering with HIF-1 activity makes for a very suitable candidate for targeting tumor metabolism.

HIF-1 is activated as a heterodimer of two subunits, a constituently activated β subunit called aryl hydrocarbon receptor nuclear translocator (Arnt) and one of three α subunits. The α subunit homologs, identified as HIF-1α, HIF-2α, or HIF-3α are regulated by O_2_ availability via induction by an O_2_-dependent post-translational modification mechanism. HIF-1α and -2α are very similar in sequence identity, and therefore have overlapping yet distinctive tissue pattern expression and target genes. HIF-3α is less understood relative to its counterparts. Importantly, HIF-1α has been demonstrated to regulate cellular metabolism in cancer cells making it a prime candidate to study as an inhibitor of increased tumor metabolism.

HIF-1α inhibitors are classified by their mechanisms of action, as there are four main targets in HIF-1α activity: targeting HIF-1α protein levels, dimerization, DNA binding, and transcription of downstream target genes. HIF-1α expression has been targeted extensively, while the other three possible mechanisms have produced fewer viable options with clinical application or promise. HIF-1α levels are shown to be increased with PI3K/AKT/mTOR activity, and PAM inhibitors are shown to decrease cellular levels of HIF-1α [64]. All of the PAM PI3K, mTOR, or PI3K/mTOR inhibitors listed in the previous section may be viable methods of suppressing tumor acidosis by lowering HIF-1α levels. The antisense oligodeoxynucleotide EZN-2968 demonstrated proof of concept viability as an inhibitor of HIF-1α in a phase I clinical trial with various refractory solid tumors, including colorectal, breast, pancreatic, adenoid cystic, mesothelioma, and Hurthle cell tumors, after showing promising results during *in vivo* experiments with 15PC3, PC3, and DU145 prostate cancer models (however, the sponsor suspended development of the agent and there are currently no active clinical trials) [65]. Additionally, topoisomerase I (Topo I) inhibitors, already commonly used as chemotherapy in the clinic, have demonstrated the ability to reduce HIF-1α expression. Specifically, topotecan reduced downstream gene expression of VEGF and GLUT in 16 patients in a clinical trial by as much as 20-fold [66]. Topotecan chemotherapy is used in the clinic to treat small cell lung cancer and overian cancer, and therefore can be a good candidate to study future HIF-1α inhibition studies in modulating tumor metabolism. A naturally occurring estrogen metabolite, 2-methoxyestradiol (2ME2) has been investigated as a phase II tubulin polymerization inhibitor in patients who have multiple myeloma, breast cancer, or prostate cancer [67]. It was shown to inhibit the nuclear translocation of HIF-1α and could also be a promising candidate for modulating aberrant metabolism in hypoxic tumors.

HIF-1α dimerization was shown to be inhibited by acriflavine, a molecule identified in an FDA approved library of drugs. In a study with a flank tumor model of PC-3 human prostate cancer, acriflavine prevented tumor growth and arrested tumor vascularization - a process that HIF-1α regulates in cancer biology [68]. Inhibiting HIF-1 from binding to DNA can be accomplished with echinomycin, a peptide that binds to the DNA recognition sequence which HIF-1α requires for transcription [69]. Bortezomib is commonly used as a proteasome inhibitor after the FDA approved its use in multiple myeloma. Bortezomib showed the ability to interfere with HIF-1α’s carboxylic acid transactivation domain by enhancing the binding of factor inhibiting HIF-1α (FIH) to HIF-1α [70]. While not as direct as targeting the protein levels of HIF-1α, these mechanisms are also possible solutions for inhibiting HIF-1α function and preventing its ability to foster an acidic microenvironment in hypoxic and highly aggressive tumors. HIF-1α activation up regulates vascular epithelial growth factor (VEGF) expression, which manifests as the angiogenic hallmark in tumor biology.

### Ion transporters

**Figure 4:**
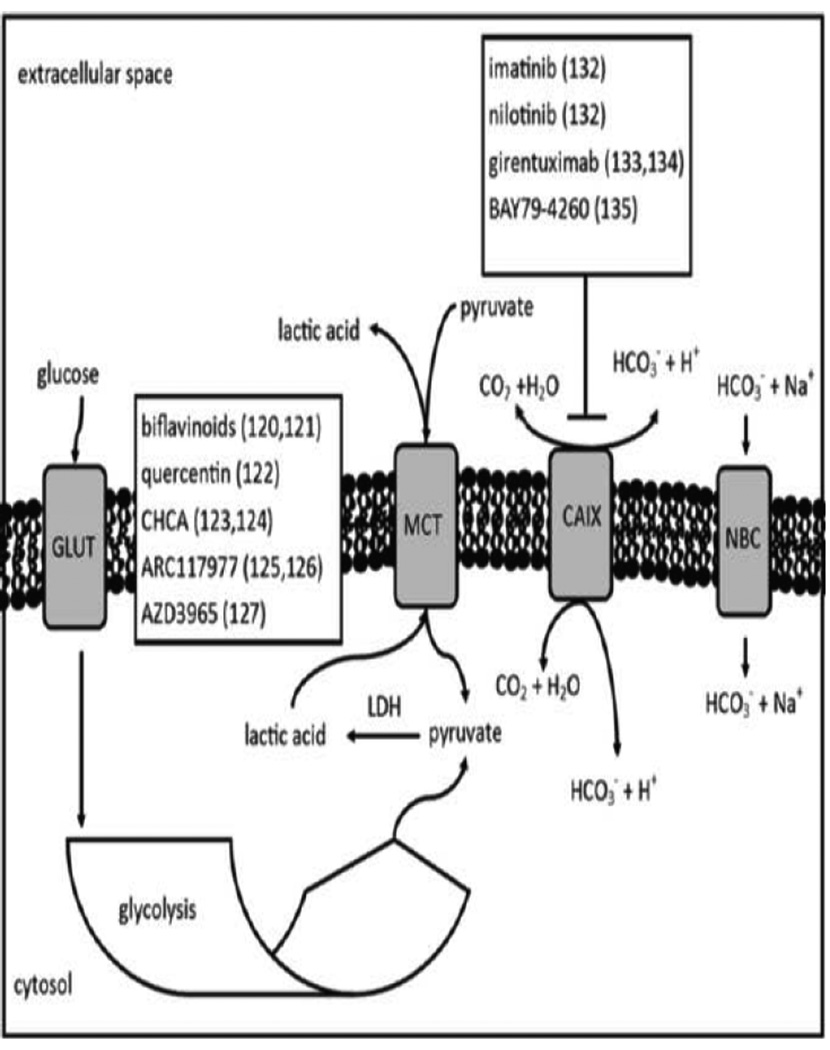
High energy metabolite transport across the membrane supports tumor metabolism balance. The monocarboxylate transporter (MCT) proteins are responsible for moving lactate and pyruvate across the membrane, supplying pyruvate for lactate dehydrogenase to convert to lactate. Lactate is one of the chief molecules responsible for acidifying the tumor extracellular space. Additionally, carbonic anhydrase IX is also known to regulate intracellular pH, acidifying the extracellular microenvironment in the process. Both MCT proteins and CAIX can be targeted by agents whose efficacy could be measured by quantifying changes in the tumor extracellular pH.

The mechanism of action of some of the molecules described in this section are not entirely tied to tumor metabolism, however the altered cellular energetics relies on their function to provide cancer cells with a sustained growth advantage. Upsetting this balance by targeting the transporters tied to these molecules could cause a cancer cell to decouple compulsory aerobic glycolytic metabolism from its energy production pathway (Figure 4). Ultimately tumor acidosis is the result of excess lactic acid in the extracellular space within the tumor microenvironment. A secondary target is the extracellular transport of lactic acid and other high energy metabolites across the membrane to the extracellular microenvironment. The monocarboxylate transporters (MCT) are a family of proteins responsible for shuttling these metabolites. Preventing the transport of lactate into the extracellular space would drive the tumor microenvironment pHe towards more neutral levels, as well as trap the lactic acid in the cytosol. The latter can be expected to have a cytoxic effect from lactic acid build up or otherwise signal for the cancer cells to switch to oxidative phosphorylation, therefore indirectly targeting tumor metabolism. Additionally, the Reverse Warburg effect describes the mechanism by which fibroblasts in the surrounding stroma produce high energy metabolites, typically lactate and pyruvate, which are then taken up by cancer cells to use for production of energy in combination with its own glycolytic cycle [71]. Therefore targeting MCTs will have the duel effect of reducing extracellular acidosis and uptake of pyruvate into the cytosol for energy production.

Monocarboxylate transporter expression patterns differ across tumors, however MCT1, MCT2, and MCT4 are frequently expressed across a variety of tumors [72]. MCT1 expression is most universal in cancer cells, and therefore makes for a prime candidate for drug targeting. Previous *in vitro* studies investigated bioflavinoids, quercentin, and α-cyano-4-hydroxycinnamic acid (CHCA) and its derivatives [73–75]. These molecules have the drawbacks of having off target effects. Some immunosuppressant compounds have been shown to be potent MCT1 blockers, such as AR-C117977 [76]. Currently the only MCT1 inhibiting agent in clinical trials is AZD3965 at its phase I stage (ClinicalTrials.gov Identifier: NCT01791595) [77].

Though the extracellular space in the tumor microenvironment is acidic compared to physiological pH, cancer cells maintain a neutral or slightly alkaline intracellular pH through the use of several other mechanisms [78]. For instance, cancers have been shown to employ carbonic anhydrase IX (CAIX) and carbonic anhydrase XII (CAXII) in hypoxic conditions [79]. CAIX hydrolizes carbon dioxide to bicarbonate and a free proton, releasing the free proton into the extracellular space and internalizing bicarbonate via the sodium bicarbonate cotransporter (NBC). This has the dual effect of both acidifying the extracellular space and neutralizing or alkalizing the intracellular space. Inhibiting CAIX function could switch the metabolism to oxidative phosphorylation as the cancer cell loses its ability to buffer the intracellular space against the acidity of lactic acid production. Known inhibitors of CAIX are imatinib and nilotinib which are extensively used in the clinic as phosphor-tyrosine kinase inhibitors [80]. The agents girentuximab and BAY79-4620 are in clinical trials as CAIX inhibitors [81,82]. Some studies of CAXII inhibitors are being investigated in leukemia models, as inhibition of CAXII activity demonstrates an increase in apoptotic cell death [83]. However, no drugs against CAXII are being actively studied in clinical trials at this time.

### Glutamine metabolism

**Figure 5:**
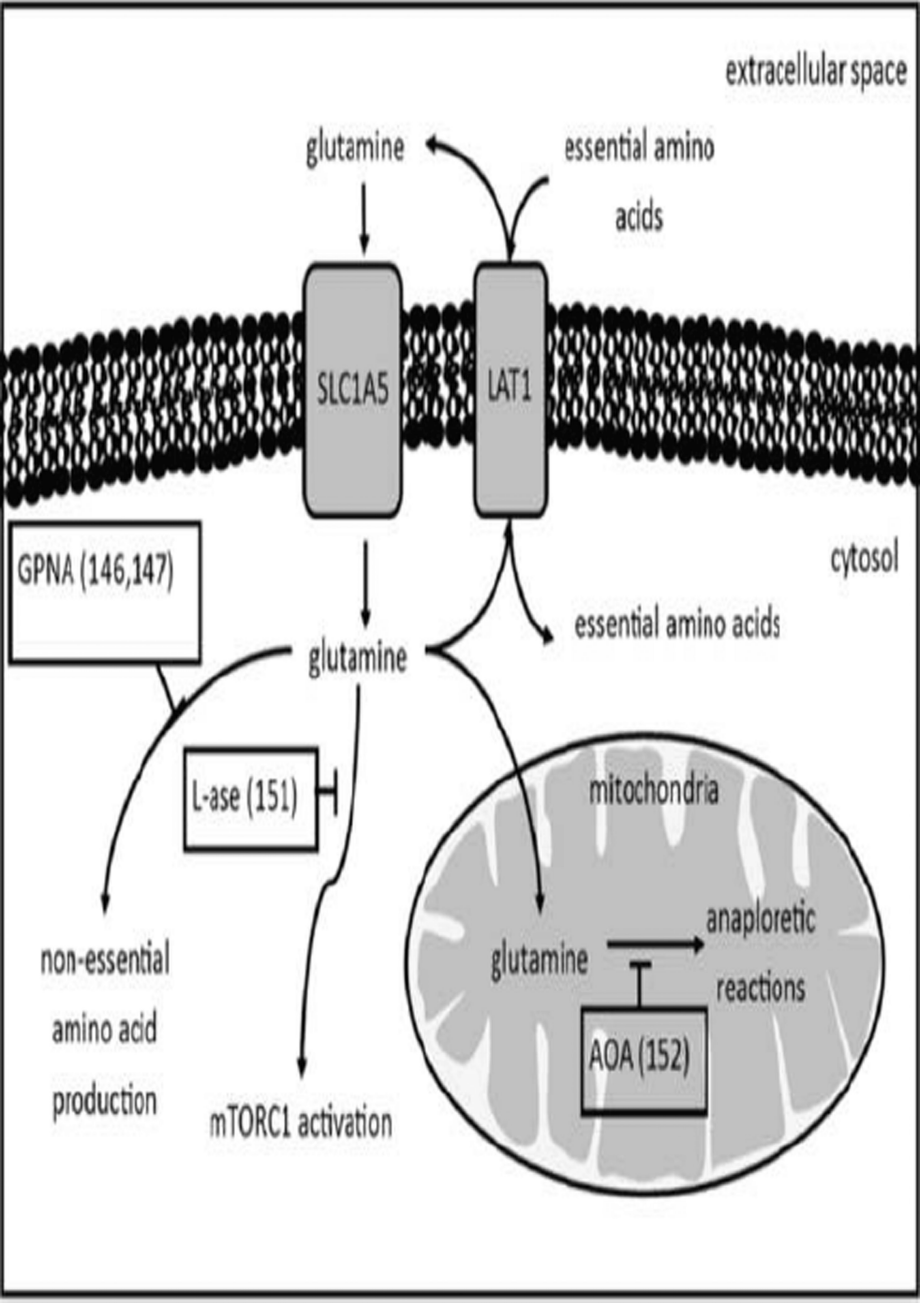
Cancer cells take up glutamine at high rates to supply carbon and nitrogen to multiple pathways in addition to protein production. Because of the plasticity of the tumor metabolome, glutamine can provide sufficient carbon for high energy metabolites in cancers. Targeting the glutamine metabolism can decrease the rate at which cancer cells produce lactic acid.

Amino acid metabolic pathways are fundamental processes in cellular growth (Figure 5). In addition to being used for protein production, amino acids feed into other pathways as metabolites or donors of nitrogen or carbon. Cancer cells take advantage of these pathways to further extend their survival advantages, such as activated PI3K/AKT/mTOR pathways, which can activate HIF-1α as previously discussed. In some cases, particular amino acid substrates or pathways have been shown to be promising targets for single agent therapies. Glutamine biology in tumor metabolism can have indirect implications in tumor acidosis as total cellular growth, and therefore energy need, is reduced. Glutamine is the most abundantly studied amino acid in tumor metabolism. While normal physiology does not use glutamine as an essential amino acid (EAA), cancer cells have been shown to rely on glutamine for anabolic growth as evidenced by increased secretion of glutamine by host tissues, transport of glutamine to the tumor cell, and increased tumor glutamine activity [84]. Termed ‘glutamine addiction,’ cancer cells use glutamine for a variety of anabolic functions, such as donating nitrogen to protein and nucleic acid synthesis, activating mTOR, and acting as a substrate in mitochondrial respiration [85–87]. Glutamine supplies nitrogen to nucleotide and non-essential amino acid (NEAA) synthesis pathways in the cell [84]. It donates its γ nitrogen in purine and pyrimidine synthesis and is converted to glutamic acid by glutaminase. The glutamic acid becomes the primary donor for nitrogen by donating its α-carbon bonded amine group in the synthesis of NEAAs. The nitrogen from the glutamic acid is transferred to various α-ketoacids produced as metabolites from glucose or glutamine, such as pyruvate. The transfer of the nitrogen to the α-ketoacids produces NEAAs. Inhibiting the nitrogen donor process would drastically slow the growth rate of cancers, as a smaller pool of amino acids lowers the rate of protein synthesis. Protein synthesis is also a one of the highest energy demanding processes in cellular biology; slowing protein synthesis in cancer may slow the energy demand and therefore slow the production of lactic acid. A study of Nγ-Aryl glutamine analogues identified a molecule, L-γ-glutamyl-p-nitroanilide (GPNA), as a glutamine uptake inhibitor [88]. Using GPNA as the foundation for a SAR study, three analogues were discovered with higher potency, although these drugs have yet to be tested *in vivo* [89].

Glutamine additionally has a role in regulating mTORC1 activity levels. Some of the intracellular glutamine is exported via the LAT1 transporter, a bidirectional amino acid transporter, in exchange for extracellular EAAs [85]. The added volume of these EAAs likely signals for mTORC1 activity. Experimental evidence has demonstrated that knockdown of the glutamine uptake transporter, SLC1A5, inhibited mTORC1 activity in acute myeloid leukemia *in vitro* [90]. As another example, the enzyme L-asparaginase (L-ase) was shown to digest glutamine, causing reduced mTORC1 activity and protein translation in an acute myeloid leukemia model [91]. As described previously, inhibiting mTOR can suppress tumor acidosis. Lastly, carbon from glutamine is shuttled directly into metabolic pathways. With the use of real time 13C NMR studies, 13C labeled glutamine was traced to the production of lactic acid *in vitro* [92]. Glutamine also supplies the carbon for anaplerotic reactions in the mitochondria, keeping the mitochondrial pool of carbon. This allows for proper mitochondrial function, both in maintaining mitochondrial membrane potential and supplying precursors for synthesis of nucleotides, proteins, and lipids. Some studies have investigated the inhibition of glutamine transamination, preventing production of metabolites such as pyruvate. The transaminase inhibitor amino oxyacetic acid (AOA) showed a reduction in tumor growth rate alone or in combination with carboplatin in subcutaneous mouse models of SUM149, SUM159, MDA-MB-231, and MCF-7 breast cancers [93].

## Imaging Tumor pHe as a Biomarker for Drug Efficacy

The current morphological imaging dogma in clinical oncology is to identify tumor masses, track changes in size, and outline surgical margins. But molecular imaging also has great potential to interrogate tumor metabolism, positioning itself as a valuable asset in a cancer biologist’s toolbox. Positron emission tomography (PET) is a prime example of molecular imaging providing information on tumor burden, but not metabolism. Using the glucose analog 2-fluorodeoxyglucose (FDG), glucose-avid tumors and secondary metastasis are routinely identified in clinical practice. Tumor ablation by chemotherapy is discernably tracked by decreases in FDG PET signals, and therefore FDG PET imaging has become a surrogate biomarker for drug efficacy. Concerning metabolism, FDG PET only measures glucose uptake, and therefore is not adept at assessing metabolic activities that employ other energy sources such as glutamine, pyruvate, and fatty acids. Tumors that adopted the Reverse Warburg effect or use glutamine or FAO to provide carbon for the TCA cycle may take up less glucose, and the faded signal in PET imaging may be a false negative result. There is a need for additional molecular imaging methods to augment FDG-PET imaging in the clinic as more tumor metabolism targeting therapies are adopted in tumor management.

**Figure 6:**
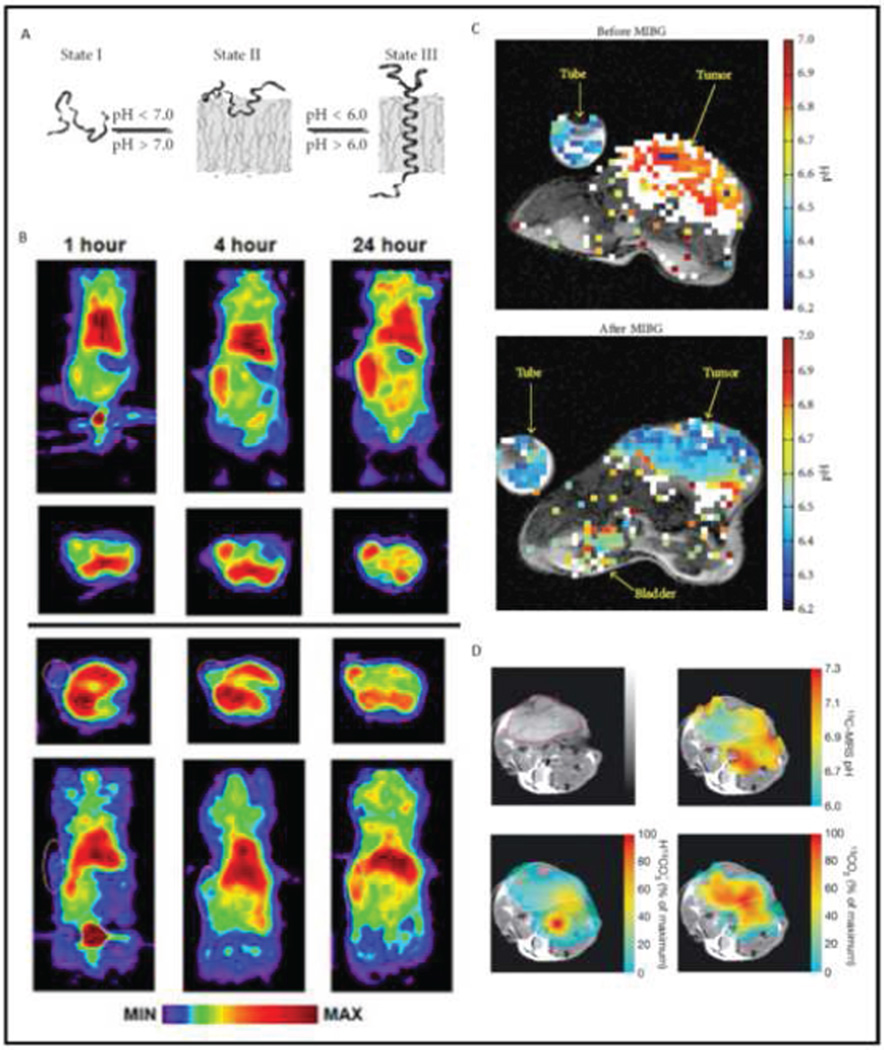
Tumor acidosis can be interrogated with noninvasive imaging. A) The pH low-insertion peptide (pHLIP) inserts itself in the membranes of cells in low pH microenvironments. B) Coupled with a 64Cu contrast agent, the pHLIP protein can be used to measure tumor pHe with positron emission topography. C) Using an FDA approved contrast agent, acidoCEST MRI measures tumor extracellular pH before and after drug treatment. D) Hyperpolarized 13C MRI can be used to measure tumor pHe by measuring the ratios of hyperpolarized bicarbonate to hyperpolarized carbon dioxide. Reproduced with permission from references 96, 99, 102, and 103.

Measuring tumor pHe can also provide clinicians with another surrogate biomarker for assessing drug efficacy. The extracellular tumor pH directly reflects the metabolic activity of the tumor by virtue of the Warburg effect. Repeatedly, studies have correlated anticancer metabolism targeted therapies with reduced growth rates or even apoptotic responses. Assessing changes in the tumor pHe during treatment is a viable method for determining drug efficacy and much sooner than detecting a reduced tumor volume with morphological imaging. The original method for determining pHe was an invasive procedure using a pH electrode [94]. However this method was very invasive and failed to take multiple readings in various loci, and therefore was not a pragmatic solution for the clinic. Several noninvasive molecular imaging methods are currently in various stages of development that are suitable for clinical translation (Figure 6).

### ^64^Cu positron emission tomography

Measuring tumor pHe with PET became possible with the advent of a ^64^Cu conjugated peptide that self inserts into cellular membranes in acidic extracellular microenvironments [95]. Using a technology called pH Low Insertion Peptide (pHLIP), this PET method had some success in preclinical models of prostate cancer by measuring acidic tumor pH values. Further improvements in sensitivity and specificity have recently been made to the technology by modifying the probe’s peptide sequence, radiometal, and chelate [96]. The method demonstrated the ability to identify a spectrum of metabolic profiles within tumors as well as discriminate between necrotic and living regions of the tumor. Tumor heterogeneity extends across all phenotypes, meaning some clones in the tumor would be more metabolically active than others. This implies a spectrum of efficacies would be observed across the clones with a single metabolism targeting strategy. Heterogeneity with respect to tumor response carries the danger of outgrowth of resistant tumor clones. Stratifying the heterogeneous acidic microenvironment could provide information on how much of the tumor can respond or has responded to a metabolism targeting agent. Knowing how much of the tumor has responded will allow clinicians to make a better informed decision on whether to continue that particular regimen or change strategies, including strategies that use a combination of drugs that also target the other pathways, allowing for more accurate personalized medicine and patient care.

### Hyperpolarized and acidoCEST magnetic resonance imaging

Several types of MRI methods are viable for measuring tumor pHe. Though typical MRI measures proton signals, instruments can be outfitted to measure ^13^C signals, allowing researchers to track carbon shuttling. The net MR signal from ^13^C at body temperature is very low, which limits the practical clinical application of ^13^C MRI or MR spectroscopy. A hyperpolarizer overcomes this limitation by increasing the ^13^C signal by as much as 10,000-fold. Hyperpolarized materials must be rapidly injected into the subject after preparation before they lose their enhanced signal strength, limiting its practicality in clinical translation [93]. Despite the complexity of hyperpolarized MRI, a MR study used hyperpolarized ^13^C tracers to measure tumor metabolism via conversion of pyruvate to lactic acid in P22 subcutaneous xenograft models in rats [97]. Additionally, ^13^C MRS was used to study the effect of the mTOR inhibitor everolimus in a lymphoma mouse model, demonstrating the ability of hyperpolarized MRI to assess drug efficacy [98]. The study saw a reduction in lactic acid production after treatment, indicating the pHe of the microenvironment may have increased. Measuring pHe directly with hyperpolarized MRI was done by taking a ratio of ^13^CHO_3_ - and ^13^CO_2_ *in vivo* [99]. A drawback to the method is that a weighted average of extracellular and intracellular pH is measured. The most direct method for imaging tumor pHe is called acidoCEST MRI which uses an FDA approved CT contrast agent to measure exchange rates of protons between the contrast agent and the bulk water of the system [100]. The contrast agents’ proton exchange rate is dependent on the concentration of hydroxide ions in the system, and is therefore able to report the pH. The method was able to accurately and precisely measure tumor pHe in lung fibrosis, breast cancer, and lymphoma in mouse models [101–103]. With either the use of iopromide or iopamidol, acidoCEST MRI makes use of enhanced perfusion in the tumor vasculature to accumulate the contrast agent in the tumor microenvironment in animal models [104]. Based on these promising results, clinical trials have been initiated that measure tumor pHe in patients who have breast, metastatic ovarian, lung, or brain cancer. Several other methodologies exist to measure tumor pHe. However their practically, and therefore suitability for use in the clinic, are limited by either engineering obstacles or complexity. For example, the use of pH-sensitive gadolinium agents measures tumor pH with T_1_-weighted MRI, but the concentration of the agent also affects T_1_-weighted MR image contrast, which severely complicates the pH measurement with these agents. In the case of the optical modalities, which measure tumor pHe by gauging shifts in the color spectrum with the use of a pH-sensitive dye, tissue imaging depth is limited by the absorbance and scattering of light [105]. Still, these optical imaging methods are used in preclinical research studies if clinical translation is not a target end point for those studies. As an example, subcutaneous xenograft models can be studied with optical fluorescence as those models have minimal tissue depth scattering [106].

## Summary

Tumor metabolism targeting therapies have the potential to interfere with tumor growth, and in combination with current chemotherapeutic strategies, inhibiting tumor metabolism could produce potent tumoricidal effects. Tumor metabolism can be targeted by inhibiting the uptake of glucose in cancer cells or its conversion to lactic acid. Additionally, tumor metabolism can be targeted more generally by inhibiting the growth signal pathways, such as the PI3K/AKT/mTOR and HIF-1 pathways. Tumor metabolism can be influenced by means of reducing the access to essential substrates for proper metabolic function, such as reducing access to glutamine. The metabolic activity of a tumor is reflected in its microenvironment as tumor acidity, and the tumor pHe can be used as a biomarker for metabolic flux. Changes in tumor pHe can be assessed by several different molecular imaging techniques, such as ^64^Cu PET-based imaging, hyperpolarized MRI, or acidoCEST MRI.
